# Comparison between Flow Cytometry, Microscopy, and Lactate Dehydrogenase-Based Enzyme-Linked Immunosorbent Assay for Plasmodium falciparum Drug Susceptibility Testing under Field Conditions

**DOI:** 10.1128/JCM.01226-15

**Published:** 2015-09-16

**Authors:** Charles J. Woodrow, Chirapat Wangsing, Kanlaya Sriprawat, Peter R. Christensen, Francois Nosten, Laurent Rénia, Bruce Russell, Benoît Malleret

**Affiliations:** a Centre for Tropical Medicine and Global Health, Nuffield Department of Medicine, University of Oxford, Oxford, United Kingdom; b Mahidol Oxford Tropical Medicine Research Unit, Faculty of Tropical Medicine, Mahidol University, Bangkok, Thailand; c Shoklo Malaria Research Unit, Faculty of Tropical Medicine, Mahidol University, Tak, Thailand; d Singapore Immunology Network (SIgN), Agency for Science, Technology and Research (A*STAR), Biopolis, Singapore; e Department of Microbiology, Yong Loo Lin School of Medicine, National University of Singapore, National University Health System, Singapore, Singapore

## Abstract

Flow cytometry is an objective method for conducting *in vitro* antimalarial sensitivity assays with increasing potential for application in field sites. We examined *in vitro* susceptibility to seven anti-malarial drugs for 40 fresh P. falciparum field isolates via a flow cytometry method (FCM), a colorimetric LDH-based ELISA **(**DELI), and standard microscopic slide analysis of growth. For FCM, 184/280 (66%) assays met analytical acceptance criteria, compared to 166/280 (59%) for DELI. There was good agreement between FCM and microscopy, while DELI tended to produce higher half-maximal inhibition constants (IC_50_s) than FCM, with an overall bias of 2.2-fold (Bland-Altman comparison). Values for artesunate and dihydroartemisinin were most affected. Paradoxical increases in signal at very high concentrations of mefloquine and related compounds were more marked with the DELI assay, suggesting that off-target effects on LDH production may be responsible. Loss of FCM signal due to reinvasion or slow growth was observed in a small number of samples. These results extend previous work on use of flow cytometry to determine antimalarial susceptibility in terms of the number of samples, range of drugs, and comparison with other methods.

## INTRODUCTION

The spread of drug-resistant Plasmodium falciparum is a public health emergency in Southeast Asia (1). The rapid and accurate detection of reduced susceptibility of P. falciparum to individual antimalarials allows action to be taken before clinical failure of a combination becomes established (2). Molecular markers of drug resistance can contribute to this process, but they rely on mechanisms of resistance being fully understood at the molecular level, and so sensitive and accurate methods for monitoring *in vitro* parasite drug sensitivity remain an important component of assessment methods (3).

For many years, *in vitro* susceptibility assays depended on microscopic analysis of blood films in the form of a readout (4). While cheap and effective, this approach has inherent problems of subjectivity and requires considerable resources in terms of training and time. The tritiated hypoxanthine radioisotope assay (5) addressed these challenges and was considered the gold standard for a considerable time (6). However, high costs and problems associated with the disposal of radioactive waste led to development of plate read antimalarial susceptibility assays, which provided a safe and cost-effective alternative. These plate read-based assays included enzyme-linked immunosorbent assays (ELISAs) on the parasite antigens lactate dehydrogenase (pLDH [7] and colorimetric LDH-based ELISA [DELI] [8]) or P. falciparum histidine-rich protein 2 (HRP2) (9) and parasite nucleic acid detection via fluorescent dyes detected in microtest format (10) or by flow cytometry (11–16). Flow cytometry methods involve the use of fluorescent dyes (e.g., SYBR green for DNA and ethidium for DNA/RNA) to stain Plasmodium DNA and RNA in malaria-infected red blood cells (iRBCs) prior to detection by a flow cytometer (17, 18). The DELI method is more sensitive than flow cytometry (0.005% of parasitemia versus 0.01% for flow cytometry assay), but results take longer to be obtained (more than a day after assay termination versus 30 min for flow cytometry). In addition to this, flow cytometry requires 5 times less volume of cell suspension than the DELI method. Importantly, flow cytometry provides a direct measure of parasite growth (with schizont maturation being the most common endpoint), whereas DELI and other ELISA methods involve the indirect measurement of parasite growth via a secondary protein marker, introducing the possibility of artifactual effects.

In this work, we carried out simultaneous *ex vivo* susceptibility tests using the DELI colorimetric microtest, flow cytometry, and microscopic analysis on fresh field isolates of Plasmodium falciparum and explored relative differences in success rate and inhibition constants for seven antimalarials.

## MATERIALS AND METHODS

### Ethics statement.

The clinical samples examined in this study were collected after ethical approval was obtained from the Oxford Tropical Research Ethics Committee (OXTREC 027-025, United Kingdom) and the ethics committee of the Faculty of Tropical Medicine, Mahidol University (MUTM 2008-215).

### Isolates and culture.

Parasite preparation, culture, and drug exposure followed the protocol described previously (18). Briefly, clinical isolates of P. falciparum were collected from patients with parasitemias of between 0.1 and 0.9%, prior to antimalarial treatment, at clinics run by the Shoklo Malaria Research Unit (SMRU) on the northwestern border of Thailand. Five milliliters of whole blood in lithium heparin collection tubes were transported to the SMRU field laboratory within 6 h of collection. Leukocyte depletion was undertaken using cellulose columns (Sigma catalog no. C6288) (19, 20), and parasitemia was adjusted to 0.1% by addition of uninfected red cells.

### Drug assay.

These assays used artesunate (AS; molecular weight [*M*_w_], 384.4 g/mol; Holly Pharmaceuticals Co. Ltd.), dihydroartemisinin (DHA; *M*_w_, 284.94; Fluka), lumefantrine (LUM; *M*_w_, 528.94; Sigma-Aldrich), mefloquine hydrochloride (MQ; *M*_w_, 414.77; Sigma-Aldrich), piperaquine tetraphosphate (PIP; *M*_w_, 999.56; Artekin Holley Pharmaceutical Co), chloroquine diphosphate (CQ; *M*_w_, 515.9; Sigma-Aldrich), and quinine hydrochloride (QN; *M*_w_, 396.9; Sigma-Aldrich). A stock solution of each drug was prepared in methanol in glass vials coated with a 0.2% (vol/vol) AquaSil water solution (Pierce) to prevent the drug from binding to the glass surface. One or two dilutions of each drug were subsequently made in methanol to obtain the drug at the desired testing concentration. Twenty microliters of the final drug solution was added in duplicate to the left-hand column of a 96-well microtiter plate (Nunc, Singapore), consisting of 87.0 nM AS, 59.6 nM DHA, 434.0 nM LUM, 3,307.9 nM MQ, 1,322.8 nM PIP, 10,256.7 nM CQ, and 12,901.4 nM QN. Ten 2-fold serial dilutions were made in methanol, and the right-hand column was left without any drug in order to measure uninhibited growth. The predosed plates were dried in an incubator at 37°C overnight, covered with Plate Sealer (Linbro), and stored at 4°C.

Four microliters of packed red blood cells at 0.1% parasitemia was mixed with 200 μl of RPMI-based culture medium and added to each well of the predosed drug plates. Plates were then incubated in an atmosphere containing 5% CO_2_ at 37°C for approximately 42 h; a slide was examined after 24 h of culture, and the duration of culture was modified in some cases according to the predicted time of schizont maturation. At the time of harvesting, the contents of wells were mixed, the contents of duplicate wells were combined into single tubes, and each resulting cell suspension was distributed appropriately in order to measure growth by three readouts (see Fig. S1 in the supplemental material).

### Colorimetric microtest (DELI).

The colorimetric microtest (DELI) was performed as described previously (8, 21) with cell suspensions frozen overnight prior to ELISA.

### Flow cytometry.

Twenty microliters of cell suspension was diluted in 80 μl of phosphate-buffered saline, stained with dihydroethidium (D7008; Sigma, Singapore) and SYBR green (10,000×; S9430; Sigma, Singapore) with 5 μg/ml and 5× as the final concentrations, respectively, and incubated for 20 min in the dark at room temperature as previously described (18). After staining, this suspension was analyzed using an Accuri C6 flow cytometer (BD Singapore). Forward scatter (FSC) and side scatter (SSC) signals of all erythrocytes were acquired via linear amplification, included potential abnormal cells due to hemoglobinopathies (22, 23). Sixty thousand events were recorded, and flow cytometry analyses were done using FlowJo software (Tree Star). The proportion of schizont events for each sample was determined via a defined gating strategy (17, 18).

### Microscopy.

Microscopic enumeration of schizont maturation was performed by a well-trained microscopist using thin blood smears stained with Giemsa with a denominator of 100 parasites.

### Calculation of IC_50_.

The growth measurements for DELI, FCM, and microscopy at each drug concentration were entered into the online IVART calculator of the WorldWide Antimalarial Resistance Network (WWARN) (24) in order to determine half-maximal inhibition constants (IC_50_s). At least 30% of parasite isolates placed in short-term culture exhibit less-than-optimal growth due to pre-exposure to drugs or other factors contributing to reduced parasite viability (6). Modified criteria to define these assays were developed based on the outputs of the nonlinear regression-based algorithm produced by the IVART analytical tool (24). IVART models a 2-parameter curve outputting IC_50_ and gamma (sigmoidicity coefficient) using the nonlinear least-squares (NLS) algorithm (25). A threshold confidence interval ratio of the IC_50_ (CIR) of <3 was selected to define core assays of higher reliability. However, the CIR parameter is not useful in a subset of assays where modeling is achieved only with a fixed gamma of 10 (the resulting 1-parameter model has artifactually narrow confidence intervals). In such cases, the growth ratio (uninhibited growth divided by maximally inhibited growth) was used to define core assays of higher reliability in accordance with previous recommendations (6, 24) by calibrating growth ratio to CIR in the larger set of 2-parameter curves. In the case of FCM, 75% of these curves with a growth ratio of 5 or more had a tight CIR (less than 3), while in this work, for DELI, this occurred with a growth ratio of 1.3 (see Fig. S2 in the supplemental material). For both FCM and DELI these rules were checked by examining the within-method correlation between MQ and QN; in both cases, these criteria produced the optimal correlation coefficient compared to cutoffs involving lower or higher growth ratios. For microscopic assays in which background is generally zero (rendering growth ratios of no value), a schizont proportion of 10% (6) rather than the growth ratio was used to define reliable 1-parameter curves.

Paradoxical growth was calculated as a proportion determined as follows: (growth at maximum drug − minimum growth)/(growth at no drug − minimum growth).

### Statistical analysis.

The median IC_50_s presented in Table 1 were compared using a Wilcoxon matched-pairs test. We used the method of Bland-Altman for assessing agreement between two methods of clinical measurement (26). Statistical analysis and graphics were carried out in Stata 12.0 (StataCorp, USA) and Prism software version 5 (GraphPad, San Diego, CA, USA).

**TABLE 1:** Geometric mean and 95% CI for seven drugs and three methods

Drug	Microscopy		Flow cytometry		Colorimetric microtest	
	*n*	Mean IC_50_ (95% CI)	*n*	Mean IC_50_ (95% CI)	*n*	Mean IC_50_ (95% CI)
AS	29	0.5459 (0.4395–0.6781)	29	0.7795 (0.565–1.075)	33	1.875 (1.487–2.363)
DHA			33	1.568 (0.9877–2.488)	28	2.653 (2.195–3.206)
LUM			23	28.25 (17.45–45.75)	22	53.89 (35.4–82.02)
MQ	35	34.48 (26.34–45.15)	21	36.29 (21.88–60.2)	18	34.22 (19.81–59.12)
PIP			26	9.131 (7.415–11.24)	29	15.12 (13.1–17.46)
CQ	36	111.2 (93.02–132.9)	30	128.5 (96.55–171)	24	189 (117.7–303.7)
QN			22	239.8 (153.4–374.7)	12	356.9 (214.5–594)

## RESULTS

### Inhibition assay of P. falciparum schizont maturation.

**FIG 1:**
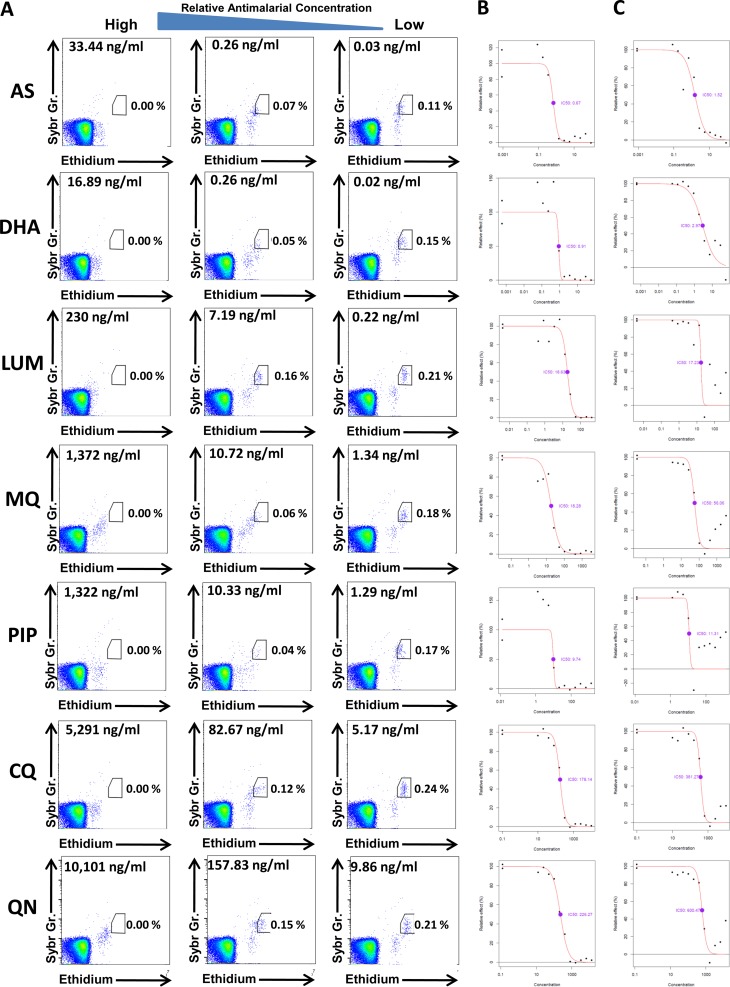
(A) Flow cytometry gating strategy to define schizont parasitemia after 42 h of drug exposure. The maturation of P. falciparum schizonts (shown in the gate) was compared to the drug-free condition. The *y* axis represents the SYBR green signal, and the *x* axis represents the ethidium signal. (B and C) Concentration-inhibition curves and IC_50_s generated with the flow cytometry (B) and DELI (C) assays.

Our flow cytometry method is based on the detection of parasite DNA (Sybr green) and RNA (ethidium) in infected red blood cells after culture in the presence of different drug dilutions (Fig. 1A) (17, 18). The schizont parasitemia is defined by cells clustered with the highest Sybr green and ethidium signals. The IC_50_s for all 7 drugs were generated by flow cytometry (Fig. 1B) and DELI (Fig. 1C) methods for 40 fresh field isolates of P. falciparum. Microscopic assessment was undertaken for AS, CQ, and MQ only. Summary IC_50_ statistics for accepted assays (see Materials and Methods) for each drug and method are presented in Table 1. The microscopic method had the highest rate of success (100/120 assays [83%]). Flow cytometry produced 184/280 (66%) accepted assays, and the colorimetric microtest (DELI) produced 166/280 (59%).

### Comparison of flow cytometry, microscopy, and DELI methods.

**FIG 2:**
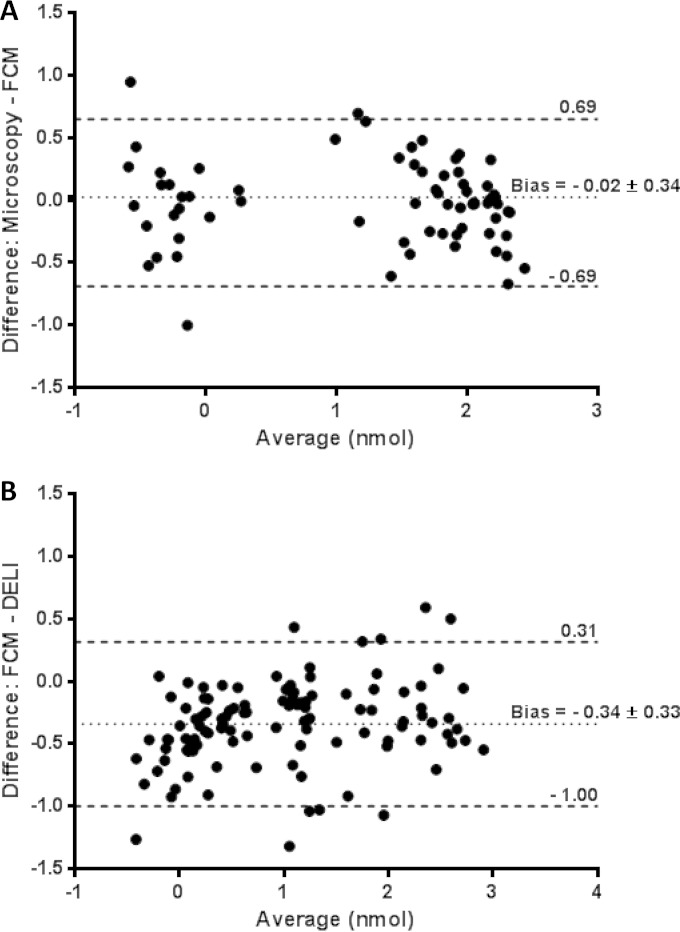
Bland-Altman plots for flow cytometry compared to microscopy (A) and DELI (B). Microscopic data were obtained for AS, CQ, and MQ, while flow cytometry and DELI colorimetric microtest data were obtained for all 7 drugs.

The summary data presented in Table 1 indicate that flow cytometry obtains IC_50_s highly analogous to those obtained by microscopy for the three drugs studied by both methods. A paired analysis of samples fulfilling acceptance criteria for both methods showed that there was no significant difference between the two methods (*P* = 0.55; Wilcoxon test). Examination by Bland-Altman analysis (26) showed good agreement across the range of samples and drugs, with a bias of less than 5% between the microscopy and flow cytometry readings (Fig. 2A).

The colorimetric ELISA (DELI) yielded higher IC_50_s than flow cytometry, with a paired analysis for samples fulfilling acceptance criteria in both methods, showing a significant difference between the two methods (*P* < 0.0001; Wilcoxon test). This was supported by Bland-Altman analysis (Fig. 2B), which showed that overall, the DELI result was around double that obtained by flow cytometry (bias of 2.2-fold). Analysis by individual drugs showed the effect was strongest with artemisinins (Table 1). As expected, paired analyses comparison of DELI with microscopy showed the same issue, with the most significant difference being found with AS (*P* < 0.0001) being smaller with CQ (Wilcoxon *P* = 0.023); no effect was observed with MQ (*P* = 0.6).

### Limitations of flow cytometry and DELI methods.

The main issues for drug inhibition assays of schizont maturation using field isolates are the variability in stage and tightness of parasite synchronization at the start of culture and the variable growth rate during culture. According to the readout method, this variability can significantly influence the final growth measurements and hence IC_50_s (6). Particular problems are seen in the following scenarios.

### (i) Low signal due to reinvasion or slow parasite growth.

**FIG 3:**
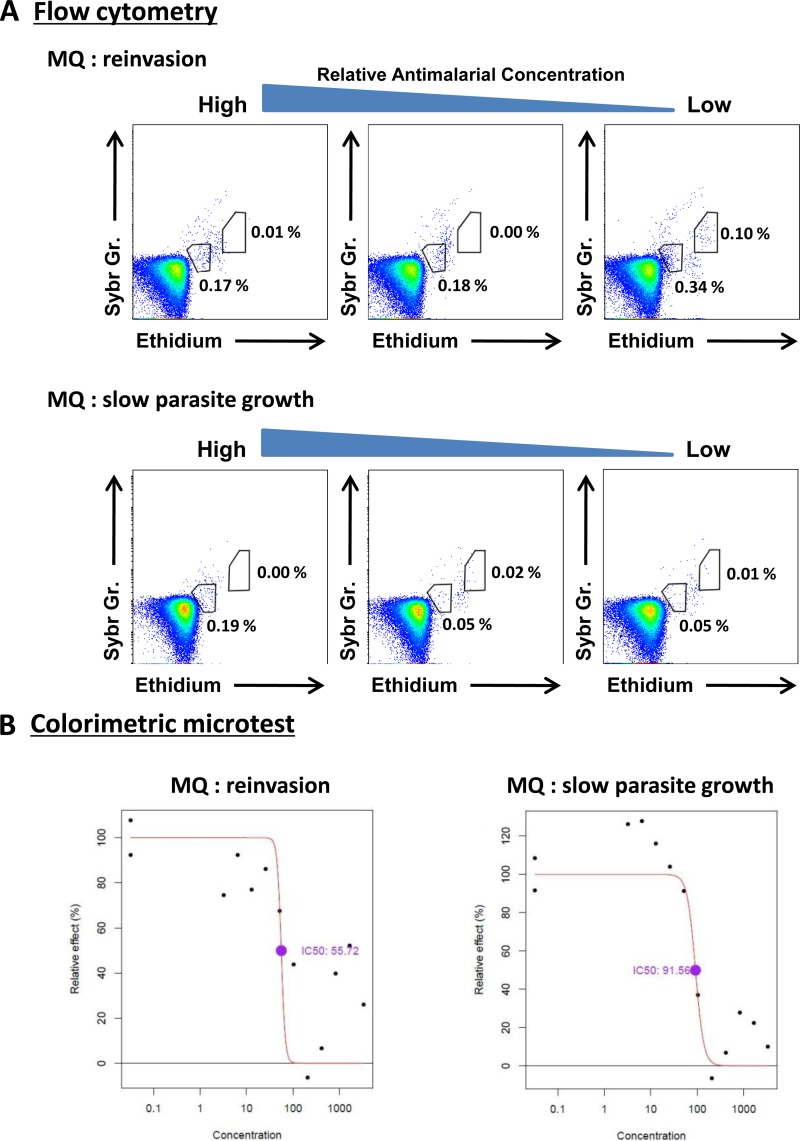
(A) Flow cytometry data illustrating two issues with P. falciparum *ex vivo* sensitivity assays, reinvasion and slow parasite growth. (B) Corresponding data for the DELI colorimetric microtest for these isolates.

One practical issue with methods relying on schizont detection (i.e., microscopy and FCM) is that if reinvasion occurs, the signal is lost. This is not a significant problem for ELISA methods, since protein simply accumulates over time and the signal is not lost at reinvasion. Although cultures were assessed at 24 h in order to predict the timing of schizogony, there were nevertheless a small number of cases where reinvasion had clearly begun the next day at the time of harvesting (Fig. 3, top), reducing signal and compromising the determination of IC_50_.

A separate issue was low parasite growth rate, in which parasite-infected cells are clustered at intermediate levels of SYBR green and ethidium fluorescence associated with the trophozoite stage (Fig. 3, bottom).

### (ii) Paradoxical increases in apparent growth at high drug concentrations.

**FIG 4:**
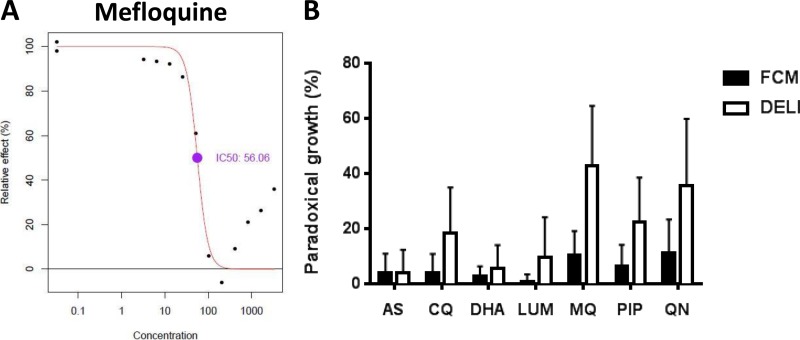
(A) An example of paradoxical increase in signal with very high concentrations of mefloquine. (B) Paradoxical increased apparent growth at high drug concentrations for the seven drugs with flow cytometry (FCM) and DELI methods (see Materials and Methods for the definition and calculation of paradoxical growth).

Previous work has noted the tendency for concentration-inhibition assays to display paradoxically increased growth at very high drug concentrations (25). A number of explanations have been put forward, including precipitation of drug at high concentrations, mixed-clone infections (27), plate edge effects (28), and “off-target” ring-stage effects (24). Because all assay readouts were derived from the same wells, it was possible to examine whether the paradoxical effect was assay method dependent. Analysis by drug and method showed that the effect was much greater in DELI assays and with nonartemisinin drugs (Fig. 4), consistent with previous observations (24). The microscopic results did not show this effect (data not shown).

## DISCUSSION

This work describes the use of flow cytometry to assess the *in vitro* sensitivity of P. falciparum field isolates, comparing the results directly to those from microscopic and ELISA methods. The development of portable flow cytometers, like the one used in this comparison, presents a viable option for running drug sensitivity assays in the field, with the added benefits of decreased sample volume and training time (29). In addition, the absence of reticulocytes in the P. falciparum drug assay limits background fluorescence due to RNA staining, unlike Plasmodium vivax studies (30, 31).

Results from the flow cytometry method (FCM) agreed closely with microscopic assessment in our field study as in previous work (17, 18), providing a rapid, objective version of the original *in vitro* gold standard method. No evidence of bias was observed, and the limits of agreement were comparable to other method comparisons of this (9, 32), although somewhat greater than in previous flow cytometry studies under field conditions (18, 33), consistent with use of a single-well flow cytometry assessment for each drug concentration.

By comparison, DELI produced substantially higher IC_50_s than microscopy or FCM. The effect clearly depended on the particular drug under study, with artemisinins and chloroquine showing the largest changes. This may reflect ring-stage production of LDH (34, 35), with higher concentrations of artemisinins being needed to inhibit ring-stage growth (36). The fact that dihydroethidium needs to be metabolized to fluoresce in viable cells could also explain this difference. FCM signal is generated in much later forms, which are more sensitive. Our findings are consistent with previous comparisons of ELISA-based assays with other methods (6, 9, 32).

The utility of different assay methods for detection of resistance to particular drugs depends on the specific properties in relation to each drug's mechanism of action (37).The production of ELISA antigens during the ring stage may hence explain why such assays have been associated with detection of progressively higher IC_50_s for artemisinins in large sample series from Cambodia (38, 39), although these assays are clearly not as focused on ring-stage assessment as the *in vitro* ring-stage survival assay (RSA) (40, 41).

By obtaining multiple readouts from the same culture wells, we were able to cast light on the cause of a long-standing issue in *in vitro* culture, that of paradoxical growth at high concentrations of nonartemisinin drugs. Overall, the evidence suggests that this may be due to an off-target effect of high concentrations of these drugs against rings and not to problems in drug solution, mixed infections, or plate effects, since the issue was specific to the LDH readout method. It is unclear why the issue appeared to be more problematic here than in previous studies of the DELI assay (8). One factor may be the difference between field isolates and culture-adapted parasites in terms of stage and growth rate, meaning that this is likely to be a specific issue for laboratories that receive parasites very soon after sampling (tiny ring stage). Another possible factor is resistance to multiple antimalarials, including artemisinins (42), which is also likely to be a particular issue in laboratories that receive parasites very soon after sampling (tiny ring stage).

In conclusion, this study illustrates that, in the context of P. falciparum drug sensitivity assays, the flow cytometry approach described here brings objectivity and speed (compared to microscopy) with lower sampling volumes and low background (compared to the colorimetric microtest (DELI), although financial and time resources in the setup phase may limit its use in field sites.

## Supplementary Material

Supplemental material
