# Supplementary material for: Comparison between Flow Cytometry, Microscopy, and Lactate Dehydrogenase-Based Enzyme-Linked Immunosorbent Assay for Plasmodium falciparum Drug Susceptibility Testing under Field Conditions
Source: J Clin Microbiol. 2015 Sep 16;53(10):3296–303. doi: 10.1128/JCM.01226-15 (PMC4572553; doi:10.1128/JCM.01226-15)
Supplement: Supplemental material [file supp_53_10_3296__index.html]

Comparison between Flow Cytometry, Microscopy, and Lactate Dehydrogenase-Based Enzyme-Linked Immunosorbent Assay for Plasmodium falciparum Drug Susceptibility Testing under Field Conditions — Supplemental material 

# Comparison between Flow Cytometry, Microscopy, and Lactate Dehydrogenase-Based Enzyme-Linked Immunosorbent Assay for Plasmodium falciparum Drug Susceptibility Testing under Field Conditions

## Supplemental material

- Supplemental file 1 -

  Fig. S1 (Schematic of experimental design for comparison between flow cytometry, microscopy, and lactate dehydrogenase ELISA) and S2 (Proportion of 2-parameter curves derived from FACS and DELI studies with wide confidence interval ratios for IC50 estimates [CIR > 3] according to different growth ratios)

  PDF, 111K
