# Supplementary material for: Comparison between Flow Cytometry, Microscopy, and Lactate Dehydrogenase-Based Enzyme-Linked Immunosorbent Assay for Plasmodium falciparum Drug Susceptibility Testing under Field Conditions
Source: J Clin Microbiol. 2015 Sep 16;53(10):3296–303. doi: 10.1128/JCM.01226-15 (PMC4572553; doi:10.1128/JCM.01226-15)
Supplement: Supplemental material [file JCM.01226-15_zjm999094532so1.pdf]

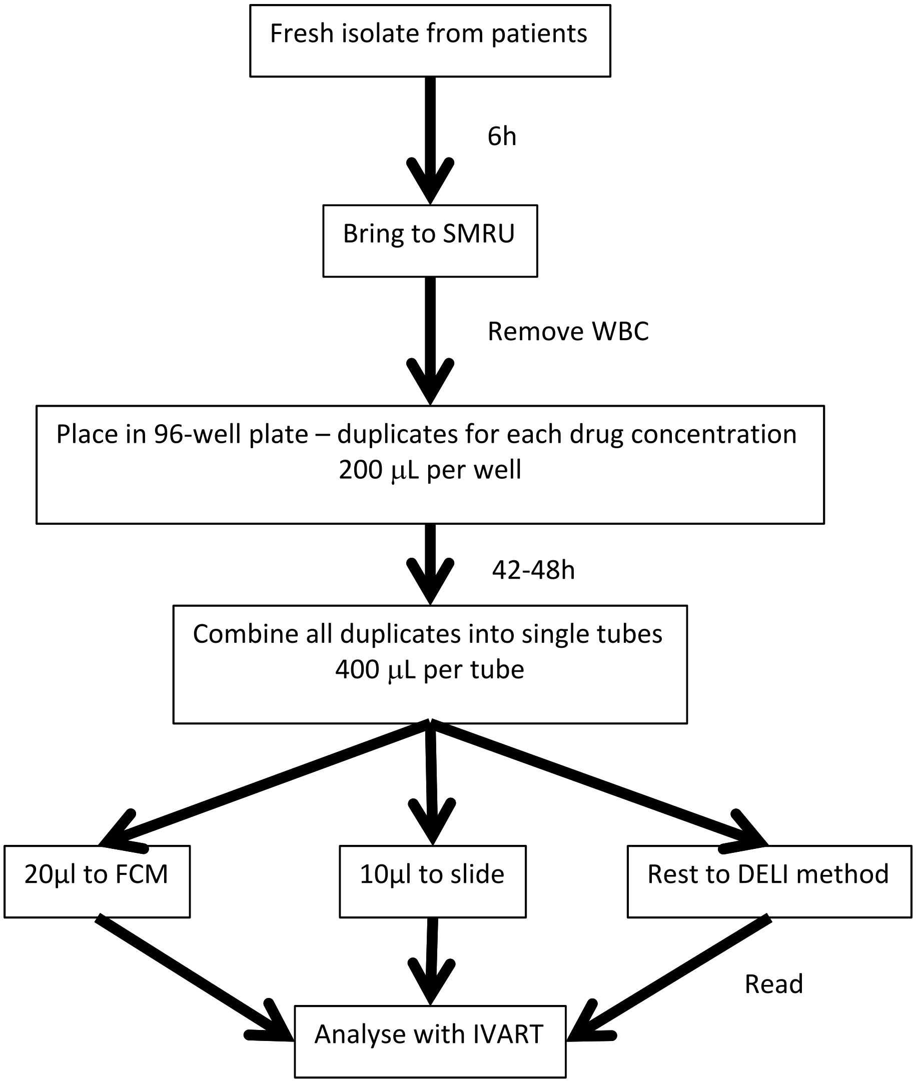

Sup. Fig. 1: Schematic of experimental design for the comparison between flow cytometry, microscopy and lactate dehydrogenase ELISA.

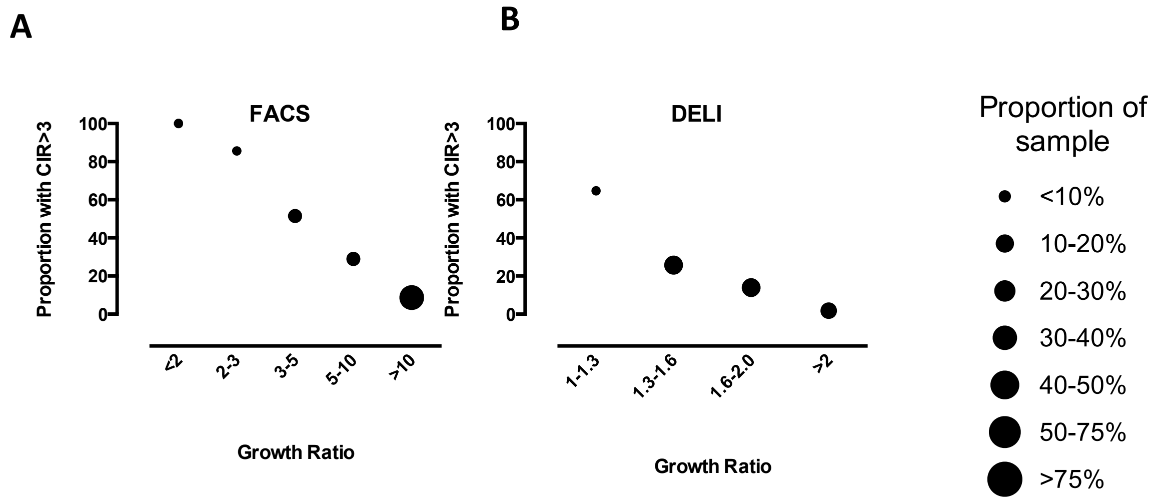

Sup. Fig. 2: Proportion of 2-parameter curves derived from FACS and DELI studies with wide confidence interval ratios for IC_{50} estimates (CIR > 3) according to different growth ratios.
